# Tuberculosis mortality and associated factors at King Abdulaziz Medical City Hospital

**DOI:** 10.1186/s12879-019-4063-7

**Published:** 2019-05-16

**Authors:** Rawabi Aljadani, Anwar E. Ahmed, Hamdan AL-Jahdali

**Affiliations:** 1 Saudi Food and Drug Authority, Riyadh, Saudi Arabia; 2 King Saud bin Abdulaziz University for Health Sciences, Riyadh, Saudi Arabia; 3 King Abdullah International Medical Research Center, Riyadh, Saudi Arabia; 4 Ministry of the National Guard-Health Affairs, Riyadh, Saudi Arabia

**Keywords:** Tuberculosis, Mortality, Multidrug-resistant

## Abstract

**Background:**

Tuberculosis (TB) continues to be a public health challenge in Saudi Arabia, particularly for the elderly. This study was conducted to estimate mortality per 1000 person-year among TB and resistant TB cases and to identifying factors associated with mortality.

**Methods:**

This is a retrospective cohort study of 713 new TB cases at King Abdulaziz Medical City in Riyadh diagnosed between January 1, 2000, and December 31, 2016. Patient medical records and microbiology lab databases were used to identify TB cases. Through reviews were conducted of patients’ medical records, including physician notes, physical examinations, radiology (scans and imaging), laboratory tests, and follow-up notes. Collected data include demographic information, clinical features, diagnoses, comorbidities, and death rates.

**Results:**

Of the 713 TB patients included in this study, 110 died, giving an average mortality rate of 22 per 1000 person-years (PY; 95% CI: 18.2–26.4). Elderly patients (≥ 60 years) had a higher mortality rate of 36.5 per 1000 PY (95% CI: 28.9–45.5). As age increases by one year, the hazard of mortality increase by 2.4% (aHR: 1.024 [95% CI: 1.009–1.039, *P* = 0.002]). Higher hazard of mortality was found among males (aHR: 2.014 [95% CI: 1.186–3.418, *P* = 0.010]). Patients with respiratory and other types of comorbidities and cancer had a higher mortality hazard (aHR: 1.898 [95% CI: 1.005–3.582, *P* = 0.048]; aHR: 2.346 [95% CI: 1.313–4.192, *P* = 0.004]; aHR: 3.292 [95% CI: 1.804–6.006, *P* = 0.001]), respectively. Multidrug-resistant TB (MDR-TB) was found in 2 cases (0.28%) (95% CI: 0.08–1.02), 1.68% were resistant to only one antibiotic, 0.14% had rifampicine-resistant TB (RR-TB), 0.28% had MDR-TB, and 0.14% had extensively drug-resistant TB (XDR-TB).

**Conclusions:**

The mortality rate among TB patients was found to be 22 per 1000 person-year at our center. TB was associated with high mortality rates among males, the elderly, and patients with cancer, respiratory illness, and other comorbidities. Future clinical practice should include establishing an efficient TB diagnostic program and continued hazard assessment of TB treatment options.

## Highlights

- This is the first study to assess mortality rate per person-years (PY) among tuberculosis patients in Saudi Arabia. The overall mortality rate among Saudi resident patients with tuberculosis was 22 per 1000 PY.
- Future clinical practice should include the hazard assessment in tuberculosis patients’ treatment plans and consider closer monitoring of cases with higher rates of mortality. In this study, higher risk of death with tuberculosis was observed among males, the elderly, cancer patients, and patients with pulmonary disease or other comorbidities.

## Background

Infectious diseases are one of the most significant public health challenges in Saudi Arabia. The concern over such diseases has played a significant role in establishing research funding priorities for pivotal institutions, such as King Abdullah International Medical Research Center, King Abdulaziz City for Science and Technology (KACST) and King Abdullah University of Science and Technology. One of these diseases is Tuberculosis (TB) which continues to be a public health concern in Saudi Arabia, particularly endangers the elderly [1]. TB mortality among Saudis was investigated from 2001 to 2010, a 6.4% fatality rate was reported, higher fatality rate was found among males and elderly [2]. Mortality from TB increases with TB resistance. TB bacteria may develop resistance to the two most powerful drugs (rifampicin and isoniazid) prescribed to treat the disease [3, 4]. This conundrum is referred to as multidrug-resistant TB (MDR-TB). In some situations, TB bacteria may develop even more severe resistance to several powerful drugs [3, 4], and this is referred to as extensively drug-resistant TB (XDR-TB). Both MDR-TB and XDR-TB are dangerous medical conditions that can affect patient outcomes, particularly mortality and TB control [5–11]. Further, TB has economic and social impacts. Economically, TB treatment ranges from $276 to $1546 USD per person for the complete treatment course. Cost estimates for MDR-TB treatment range from $1000 to $10,000 USD [12]. Additionally, treatment complications and hospitalization may increase indirect costs. Socially, TB affects all quality of life domains, including general health perceptions, psychological health, well-being, and social functioning [13]. The World Health Organization (WHO; 2015) recently reported an estimated 580,000 cases of MDR/rifampicin-resistant TB (MDR/RR-TB), with MDR-TB accounting for 83% [11]. In 2015, there were about 250,000 deaths from MDR/RR-TB, worldwide [11]. Concerning Saudi Arabia, WHO has reported an annual estimate of 150 (range, 120–180) cases with a rate of 0.48 per 100,000 of MDR/RR-TB [14]. Many studies have investigated the prevalence of TB in Saudi Arabia. A retrospective review of microbiology and infection control databases at King Faisal Specialist Hospital and Research Centre reported an overall anti-TB drug resistance rate of 2.5% in 764 TB isolates [15]. A nationwide study from seven regions of Saudi Arabia reported a rate of 4.5% in 1505 clinical isolates of TB [16]. A study in a Saudi tertiary care unit on 289 isolates reported at least one anti-TB drug (8.7%) [17]. Another study reported an MDR-TB rate of 1.4% in a sample of antibiotic susceptibility of 1681 non-repetitive TB isolates [18]. Nonetheless, mortality and risk factors associated with TB and MDR TB are not well studied in Saudi Arabia, none of the previous studies assessed TB mortality rate per person-years (PY) among the Saudi population. In this study, we aim to explore the mortality rate per person-years (PY) of TB and resistant TB patients and identify the potential risk factors associated with higher mortality, which is crucial in TB control and treatment outcome improvements.

## Methods

This is a retrospective cohort study of new TB cases at King Abdulaziz Medical City Hospital between January 1, 2000, and December 31, 2016. A list of all registered confirmed or suspected TB patients during the prespecified timeframe was obtained from the medical records and microbiology lab databases. Next, full medical records for all of the patients were thoroughly reviewed (including physician notes, physical examinations, radiology (scans and imaging), laboratory tests, and follow-up notes), and newly diagnosed TB cases during the study timeframe were identified. For the last 16 years, King Abdul Aziz Medical City stored all patients’ medical history in paper-based health documents. TB was diagnosed according to the American Thoracic Society/Infectious Diseases Society of America/Centers for Disease Control and Prevention Clinical Practice Guidelines [19]. Pulmonary TB (PTB) was diagnosed based on the symptoms, chest X-rays, and laboratory testing results of either a three acid-fast bacilli (AFB) smear and/or QuantiFERON-TB Gold test. Extra pulmonary TB (EPTB) was diagnosed based on symptoms, radiological scans results, and the results of suspected location biopsy or aspirate culture. Positive specimens were tested for drug susceptibility for rifampicin, isoniazid, ethambutol, and streptomycin to identify resistant cases. Both sexes and all age groups were eligible for inclusion.

Data were collected from the medical records using a structured data collection form. Demographic data included age, gender, nationality, smoking status, and body mass index (BMI). Clinical data included general (diarrhea, nausea, vomiting), systemic (fever, headache, chest pain), respiratory (cough, hemoptysis, shortness of breath), and lung cavitations. We also recorded the following patient diagnosis and comorbidity data: TB status, TB site, drug resistance, respiratory comorbidities (pulmonary disease, asthma, chronic obstructive pulmonary disease), other comorbidities (diabetes, hypertension, heart disease, hyperlipidemia, liver disease, renal disease), cancer, immunosuppression due to infection, genetic condition, and medication. In addition, we collected the following data about death: date of death, date of TB diagnosis, and date of loss to follow up (date of last follow-up visit to the clinic). TB was considered the cause of death in all patients who died while under anti-TB treatment, regardless of the primary cause of death. Person-years (PY) of cohort members started when anti-TB treatment was initiated and ended on the date of death, date of loss to follow up, or December 31, 2016, whichever came first. This research was funded by KACST Grant Programs for Universities and Research Centers (GPURC).

### Statistical analysis

Descriptive statistics were used to summarize patient characteristics; Frequencies and percentages (n, %) were computed to describe and summarize categorical variables. We calculated mortality rates per 1000 PY and 95% CIs. The Cox proportional hazards (CPH) model was used to identify risk factors associated with shorter survival among TB cases. In all analyses, *P* < 0.05 was considered significant. Databases were analyzed using SPSS version 23 [20].

## Results

In this study, 713 patients were diagnosed with TB at King Abdulaziz Medical City Hospital between January 1, 2000, and December 31, 2016. The majority were male (58.80%), Saudi (93.7%), and aged 30 years or older. Of the patients, 13.7% were obese, and 53.6 and 55.4% complained of respiratory and systemic symptoms, respectively. Of the sample, 9.7% had respiratory comorbidities, 6.6% had cancer, and 49.9% had pulmonary TB. Other characteristics are mentioned in Table 1.

**Table 1:** Patient’s characteristics

Characteristics	Levels	*N*	%
Age years (1–101)	≤14	39	5.5
	15–29	114	16
	30–59	240	33
	≥60	320	44.9
Gender	Male	419	58.8
	Female	294	41.2
Nationality	Saudi	668	93.7
	Non-Saudi	45	6.3
BMI kg/m^2^ (11.12–53.41)	< 18.5	100	20.4
	18.5–24.9	202	41.3
	25–29.9	120	24.5
	≥ 30 (Obesity)	67	13.7
Smoker		40	6.2
Clinical features	General	85	11.9
	Systemic	395	55.4
	Respiratory	382	53.6
	Cavitations	91	12.8
Site of TB	Pulmonary	356	49.9
	Extra Pulmonary	357	50.1
Resistant		19	2.7
Comorbidities	Respiratory	69	9.7
	Other	377	52.9
	Cancer	47	6.6
	Immunocompromised	47	6.6

*BM* Body mass index

In our study, 19 (2.66%; 95% CI: 1.71–4.12) had resistant TB, and MDR-TB was found in 2 cases (0.28%; 95% CI: 0.08–1.02). Additionally, 1.68% were resistant to only one antibiotic, 0.14% had RR-TB, 0.28% had MDR-TB, and 0.14% had XDR-TB. Isoniazid and streptomycin had the highest resistance rates of 1.68 and 1.4%, respectively. Antibiotics resistant cases are summarized in Table 2.

**Table 2:** Antibiotics resistant cases

Resistant cases	Antibiotic	*N*	%
Resistant to only one antibiotic	Streptomycin	6	0.84
	Isoniazid	5	0.7
	Rifampicin (RR-TB)	1	0.14
Resistant to two antibiotic	Isoniazid + Streptomycin	3	0.42
	Isoniazid + Ethambutol	1	0.14
	Isoniazid + Rifampicin (MDR-TB)	2	0.28
Resistant to all antibiotic	XDR-TB	1	0.14

*RR-TB* rifampicin-resistant tuberculosis, *MDR-TB* multidrug-resistant tuberculosis, *XDR-TB* Extensively drug-resistant tuberculosis

**Fig. 1:**
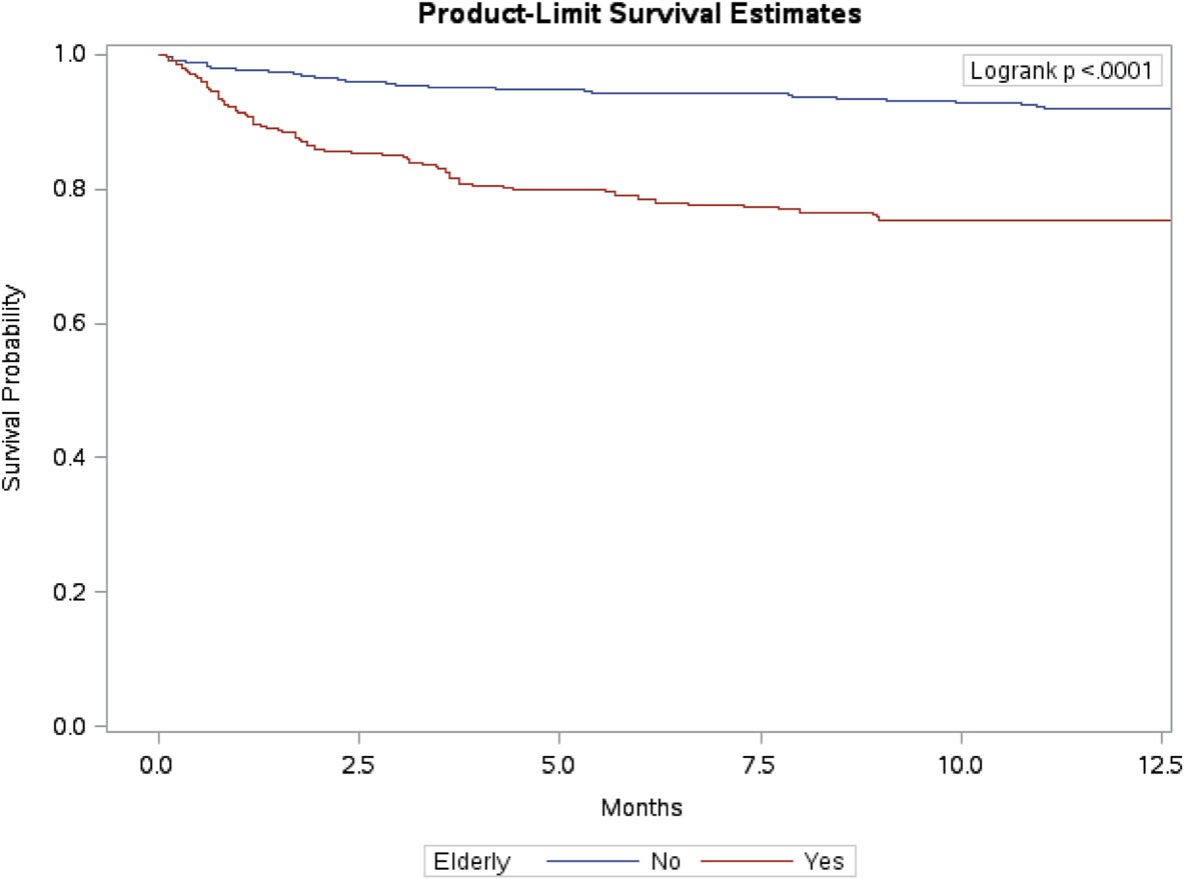
Survival probability stratified by age groups

**Fig. 2:**
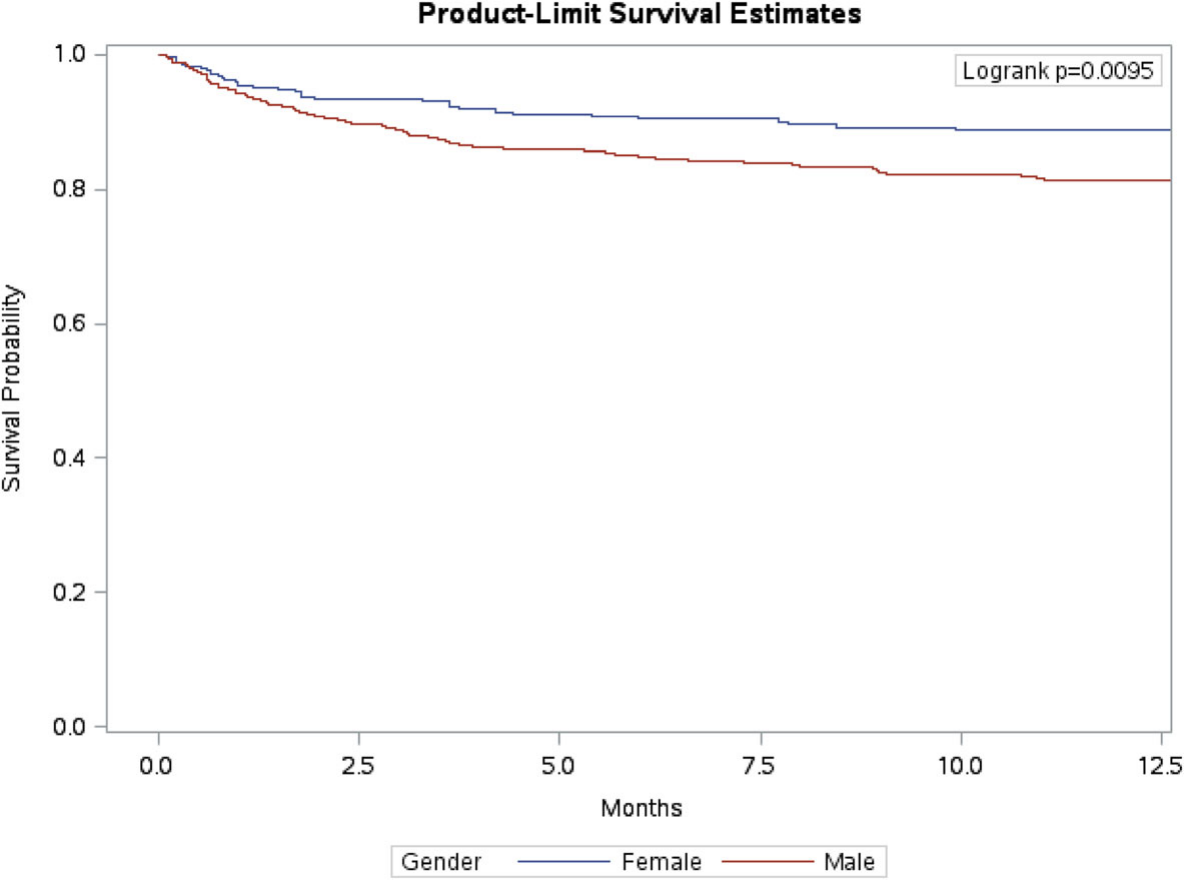
Survival probability stratified by gender

Mortality rates (per 1000 PY) are summarized in Table 3. Of the 713 TB patients included in this study, 110 died with an active TB, giving an average mortality rate of 22 per 1000 PY (95% CI: 18.2–26.4). Elderly patients 60 years old and above had a mortality rate of 36.5 per 1000 PY (95% CI: 28.9–45.5), with survival probability stratified by age group, as illustrated in Fig. 1. Males had a mortality rate of 28.4 per 1000 PY (95% CI: 22.5–35.3), with survival probability stratified by gender, as illustrated in Fig. 2. Mortality rate was the highest among PTB patients at 126.8 per 1000 PY (95% CI: 97.2–162.8). Patients with respiratory comorbidities and patients with other comorbidities had mortality rates of 33.7 per 1000 PY (95% CI: 20.27–52.83) and 31.7 per 1000 PY (95% CI: 25.25–39.28), respectively. Cancer patients with TB had a mortality rate of 84.3 per 1000 PY (95% CI: 52.2–129.2).

**Table 3:** Mortality rates (per 1000 person-years (PY)) in NGHA (2000–2016)

	Level	Person-years	Deaths	Rate	95% CI	
					Lower	Upper
Overall		4990.97	110	22.0	18.2	26.4
Age years	≤14	275.06	3	10.9	2.7	29.6
	15–29	773.43	6	7.8	3.1	16.1
	30–59	1872.26	26	13.9	9.2	20
	≥60	2054.16	75	36.5	28.9	45.5
Gender	Male	2676.86	76	28.4	22.5	35.3
	Female	2314.11	34	14.7	10.3	20.3
Nationality	Saudi	4760.43	102	21.4	17.5	25.9
	Non-Saudi	230.54	8	34.7	16.1	65.9
Obesity	Yes	2669.93	12	4.4	2.4	7.6
	No	2321.04	67	28.9	22.5	36.4
Smoke	Smoker	255.07	5	19.6	7.1	43.4
	Non-smoker	4065.54	102	25.1	20.5	30.3
Clinical features	General	507.54	18	35.5	21.6	54.9
	Systemic	2941.69	56	19.0	14.52	24.54
	Respiratory	2871.86	55	19.2	14.5	24.7
	Cavitations	713.69	9	12.6	6.1	23.1
Site of TB	Pulmonary	457.21	58	126.8	97.2	162.8
	Extra Pulmonary	2807.25	52	18.5	13.9	24.1
Resistant	Yes	135.8	1	7.4	0.3	36.3
	No	4855.71	109	22.4	18.5	26.9
Comorbidities	Respiratory	504.76	17	33.7	20.27	52.83
	Other	2493.39	79	31.7	25.25	39.28
	Cancer	225.43	19	84.3	52.2	129.2
	Immunocompromised	344.73	8	23.2	10.7	44

The results of the univariate and multivariate CPH models are included in Table 4. The univariate CPH model shows that as age increased by one year, mortality hazard increased by 3.1% (HR: 1.031 [95% CI: 1.021–1.042, *P* = 0.001]). The hazard of mortality was 69.7% higher in males (HR: 1.697 [95% CI: 1.132–2.542, *P* = 0.010]). Patients with respiratory comorbidities had 82.3% higher hazard (HR: 1.823 [95% CI: 1.087–3.057, *P* = 0.023]). Patients with other types of comorbidities has two times higher mortality hazard (HR: 2.377 [95% CI: 1.569–3.602, *P* = 0.001]). The hazard of mortality was three times higher in cancer patients (HR: 3.274 [95% CI: 1.995–5.373, *P* = 0.001]). According to the results of the multivariate CPH model, as age increased by one year, the hazard of mortality increased by 2.4% (aHR: 1.024 [95% CI: 1.009–1.039, *P* = 0.002]). The hazard of mortality was higher among males (aHR: 2.014 [95% CI: 1.186–3.418, *P* = 0.010]). Patients who presented with general symptoms had 91.3% higher hazard of mortality (aHR: 1.913 [95% CI: 1.004–3.644, *P* = 0.049]). Patients with respiratory comorbidities, other type of comorbidities, and cancer had higher mortality hazards (aHR: 1.898 [95% CI: 1.005–3.582, *P* = 0.048], aHR: 2.346 [95% CI: 1.313–4.192, *P* = 0.004], aHR: 3.292 [95% CI: 1.804–6.006, *P* = 0.001]), respectively.

**Table 4:** Factors associated with 5-year mortality rate among TB cases

Variables		Univariate			Multivariate			
	*P*	HR	95% CI		*P*	aHR	95% CI	
			Lower	Upper			Lower	Upper
Age	0.001*	1.031	1.021	1.042	0.002*	1.024	1.009	1.039
Male	0.010*	1.697	1.132	2.542	0.010*	2.014	1.186	3.418
Saudi	0.296	0.681	0.332	1.4	0.282	0.599	0.235	1.525
Obesity	0.816	1.076	0.582	1.989	0.677	0.87	0.452	1.676
Smoke	0.579	0.776	0.316	1.904	0.912	1.055	0.41	2.712
Clinical features								
General	0.092	1.545	0.932	2.56	0.049*	1.913	1.004	3.644
Systemic	0.248	0.802	0.552	1.166	0.234	0.747	0.462	1.208
Respiratory	0.372	0.843	0.58	1.225	0.796	1.074	0.627	1.837
Cavitations	0.093	0.558	0.282	1.103	0.064	0.441	0.186	1.049
Site of TB	0.396	1.176	0.809	1.71	0.744	1.093	0.641	1.861
Comorbidities								
Respiratory	0.023*	1.823	1.087	3.057	0.048*	1.898	1.005	3.582
Other	0.001*	2.377	1.569	3.602	0.004*	2.346	1.313	4.192
Cancer	0.001*	3.274	1.995	5.373	0.001*	3.292	1.804	6.006
Immunocompromised	0.842	1.076	0.524	2.209	0.319	0.589	0.208	1.668

*Wald chi-square test is significant α = 0.05. HR, hazard ratio; aHR, adjusted hazard ratio

## Discussion

We found an overall mortality rate of 22 per 1000 PY, with higher mortality rates among the elderly, males, and patients with PTB. Also, we found higher mortality hazard among patients with respiratory comorbidities, other type of comorbidities, and cancer. A retrospective cohort study found a mortality rate of 8.79 per 1000 PY [21]. The difference between these findings could be explained by the underlying differences in populations under investigation, trends of TB, and patterns of TB spread among the population. Many other studies have investigated TB mortality [2, 22–25], but, unfortunately, direct comparison between our results and their results cannot be made due to different estimates used to assess mortality among these populations. Most of the studies used case fatality rates (CFRs) and standardized death ratio (SMR), while in our study we used mortality rate per PY.

Our study demonstrated a higher mortality rate among the elderly. We found a positive association between age and the hazard of mortality, which is consistent with several studies [2, 26, 27], the first of which is a Saudi cohort reporting a positive correlation between age and mortality among TB patients as well. The highest mortality rate among age groups was in the elderly (≥ 65 years) [2]. The second study evaluated mortality in a large TB treatment trial conducted in the United States and Canada, which found a similar hazard of mortality with age [26]. The last study, which included Taiwanese TB patients, also reported a higher hazard of mortality in age group of ≥75 [27]. Males are more prone to death from TB. They have a double mortality rate and 90% higher hazard compared to females, which agrees with the results of previous studies [2, 22, 23, 28, 29]. An analysis of European surveillance data in 2008 showed a 50% higher risk of death among male TB patients [30]. The positive association between males and death has also been reported in many other studies [24, 25, 31]. The highest mortality rate we found was among PTB patients. Another study of 3451 patients also reported a higher mortality rate among PTB patients compared to extra-pulmonary [32].

The hazard of death was 89.9% and two times higher among patients with pulmonary and other comorbidities. We did not find many studies investigating the differences between the effect of these two comorbidities on TB mortality, rather they studied the effect of the presence or absence of chronic diseases in general without specification. One study reported a three times higher hazard of death among patients with at least one comorbidity [28]. Cancer patients had the second highest mortality rate; the hazard of death was four times higher among cancer patients with TB, and similar findings were reported by several researchers. A higher risk of TB patient death was associated with malignancy in several studies [26, 33].

This is a retrospective cohort study that has several limitations regarding data quality. It is a single-center experience with a small number of identified resistant cases and no MDR-TB reporting system to ensure accuracy of cases identifications. We investigated TB mortality in a tertiary care hospital setting, which tends to have patients with more advanced TB disease condition and co-multi-morbidities. Data about human immunodeficiency virus (HIV) infection was unavailable, although we collected data about immunosuppression in general. Socioeconomic data were also unavailable. Further multicenter studies are needed to accurately estimate the mortality rate of resistant TB in this population and explore other potential risk factors associated with TB mortality, including human immunodeficiency virus (HIV) infection and socioeconomic factors, such as income.

## Conclusions

Mortality rate among TB patients was found 22 per 1000 PY in our center. TB was associated with high mortality rate among males, the elderly, and patients with cancer, respiratory, and other comorbidities. Future clinical practice should include establishing efficient TB diagnostic programs and continued hazard assessments in TB patients’ treatment options. Extra caution must be taken while treating the elderly and patients with one or more comorbidities. Additionally, clinicians should be alert to any sign of treatment failure.

## Abbreviations

AFB: Acid-Fast Bacilli
aHR: Adjusted Hazard Ratio
BMI: Body Mass Index
CFRs: Case Fatality Rates
CPH: Cox Proportional Hazards
EPTB: Extra Pulmonary Tuberculosis
HR: Hazard Ratio
MDR-TB: Multidrug-Resistant Tuberculosis
PTB: Pulmonary Tuberculosis
PY: Person-Years
RR-TB: Rifampicin-Resistant Tuberculosis
SMR: Standardized Death Ratio
TB: Tuberculosis
WHO: The World Health Organization
XDR-TB: Extensively Drug-Resistant Tuberculosis
